# Customer Baseline Credibility in Constrained Reinforcement Learning for Incentive-Based Demand Response

**DOI:** 10.3390/s26133986

**Published:** 2026-06-23

**Authors:** Jiyong Li, Kaiyue Wang

**Affiliations:** Department of Electrical Engineering, Guangxi University, Nanning 530004, China

**Keywords:** incentive-based demand response, customer baseline credibility, constrained reinforcement learning, resource allocation, action correction, incentive settlement, renewable energy accommodation

## Abstract

Incentive-based demand response is an important flexibility resource for power systems with high-renewable energy penetration. However, practical incentive allocation depends not only on flexible capacity and user response uncertainty, but also on the credibility of customer baseline load (CBL), which directly affects response measurement, verification, and incentive settlement. To address this issue, this paper proposes a constrained reinforcement learning method with customer baseline credibility for dynamic resource allocation in incentive-based demand response. Based on user-side load measurements and demand response event records, the proposed framework evaluates user resources using flexible capacity, response reliability, response cost, and CBL credibility. The CBL credibility score reflects the measurement quality of the delivered response and is used as a pre-event allocation factor. Users are then grouped into different resource levels, and a group-level reinforcement learning agent dynamically determines incentive multipliers and response task allocation ratios. To improve feasibility, an action correction module revises raw policy outputs under budget, price, response capacity, and CBL risk constraints before implementation. Case studies are conducted using public industrial demand response measurements and open electricity-system time-series data. The results show that the proposed CBL-CRL method reduces the normalized total operating cost to 0.897, reduces the response tracking error to 0.108, and lowers CBL risk exposure to 0.087 under the normal scenario. Relative to the No-DR reference, CBL-CRL reduces the normalized total operating cost by 10.3 percent. Compared with MAPPO, the strongest learning-based baseline, CBL-CRL reduces the response tracking error by 10.7 percent and the CBL risk exposure by 40.8 percent, while maintaining the same renewable accommodation rate of 0.970. Compared with rule-based and learning-based baselines, CBL-CRL achieves a better balance between operational performance, incentive efficiency, action feasibility, and baseline-related settlement reliability. The results demonstrate that CBL credibility should not only be used for post-event settlement, but can also serve as an effective pre-event resource allocation factor for measurement-driven demand response programs.

## 1. Introduction

The large-scale integration of renewable energy is changing the operational structure of modern power systems. Wind and photovoltaic generation reduce carbon emissions, but their variability and uncertainty also increase the difficulty of maintaining supply–demand balance. As a result, power systems increasingly require flexible resources that can respond to short-term net-load fluctuations, peak-load periods, and renewable generation surplus. Demand response has become one of the most important demand-side flexibility resources because it can reshape electricity consumption without relying only on generation-side reserves or long-cycle infrastructure investment. Previous studies have shown that demand-side flexibility can contribute to peak-load reduction, renewable energy accommodation, backup-capacity reduction, and lower system flexibility costs [1,2]. Recent studies further indicate that demand flexibility can play a significant role in renewable integration and urban flexibility planning under high-renewable scenarios [3,4].

Demand response has evolved from a simple load-reduction measure into a system-level flexibility resource supported by smart grid technologies, advanced metering, communication systems, and automated control. In liberalized electricity markets, demand-side flexibility is increasingly expected to participate in energy balancing, congestion management, ancillary services, and capacity-related mechanisms. However, the operational value of demand response depends not only on the amount of flexible load that can be aggregated, but also on whether such flexibility can be reliably observed, verified, dispatched, and settled. This makes demand response a joint problem of resource identification, behavioral modeling, incentive design, and market coordination [5,6]. Recent studies on AMI-based short-term forecasting and IoT-enabled building energy management also show that data acquisition, sensing infrastructure, and automated control are important foundations for implementing reliable demand response programs [7,8]. Lightweight recognition networks and time-series anomaly detection methods further show that reliable sensing-data processing is important for extracting useful information from heterogeneous measurements [9,10]. Recent discussions on demand-side flexibility governance and market design further suggest that scalable demand response requires both technical feasibility and institutional credibility [11,12].

Among different demand response mechanisms, incentive-based demand response is particularly suitable for situations where system operators or aggregators require a more controllable response target. Incentive-based programs provide explicit compensation to users who adjust their consumption during specified events. Early studies investigated incentive-compatible contracts, real-time response models, and user-side scheduling mechanisms, laying the foundation for incentive design in demand management [13,14]. More recent studies have extended incentive-based demand response and related demand-side flexibility scheduling to privacy-preserving optimization, integrated energy systems, carbon-related operation, consumer-constrained pricing schemes, industrial-load coordination under high wind-power integration, IoT-enabled building energy management [8,15,16,17,18], generalized-energy-storage-based multi-timescale scheduling [19], and data-center computation–electricity co-scheduling with flexible batch loads [20]. At the equipment and microgrid level, voltage-balancing converters, fault-tolerant voltage balancers, and high-reliability multi-input converters also support reliable operation of distributed-energy and DC power systems [21,22,23]. These studies show that incentive design is no longer a static pricing problem, but a dynamic resource allocation problem involving heterogeneous users, limited budgets, and system-level flexibility requirements.

A major challenge in incentive-based demand response is user heterogeneity. Different users respond differently to the same incentive because they differ in production processes, comfort preferences, appliance ownership, response delay, risk perception, and historical participation experience. Behavioral studies have shown that user response cannot be fully explained by economic reward alone; cognitive processes, perceived risk, convenience, and contextual factors also influence participation decisions [24]. In building and appliance-level demand response, the actual flexibility of users is shaped by device-level controllability, building operating conditions, and the availability of automated control interfaces [25,26]. Recent data-driven studies further suggest that demand response potential can be better identified by mining micro-behavioral patterns and heterogeneous user features [27,28].

In addition to user heterogeneity, measurement and verification are crucial for incentive-based demand response. Customer baseline load is the reference used to estimate what a user would have consumed if no demand response event had occurred. The delivered response is then calculated by comparing the baseline with the measured event-period load. If the baseline is overestimated, users may obtain excessive compensation without providing real flexibility; if it is underestimated, users who genuinely reduce load may be underpaid. Therefore, customer baseline load is not merely a load forecasting result, but a settlement reference that affects fairness, transparency, and market credibility. Earlier work treated baseline estimation as a measurement and verification problem [29]. Recent studies have improved baseline estimation and event-period load prediction using attention-based generative reconstruction, adaptive federated learning, AMI-based short-term forecasting, electric-vehicle-oriented baseline analysis, and physics-informed high-frequency modeling [7,30,31,32,33]. A recent review also emphasizes that baseline estimation in incentive-based demand response is closely related to accuracy, interpretability, transparency, and resistance to manipulation [34]. These studies make CBL estimation more accurate and auditable, but their main role is still to support response measurement and settlement after an event. They do not directly answer how baseline credibility should affect the aggregator’s pre-event selection of users and allocation of incentive tasks.

Despite these advances, most baseline-related studies still use customer baseline load mainly as a post-event accounting tool. In a typical implementation process, the aggregator first decides which users to call and how much incentive to offer, and only after the event uses the baseline to calculate actual response and compensation. This sequential treatment overlooks an important operational issue: baseline credibility differs across users and can directly affect the quality of incentive allocation. Users with unstable historical load profiles may appear to have considerable flexible capacity, but their actual response may be difficult to verify reliably. If such users are overused, the program may suffer from settlement errors, inefficient incentive expenditure, and disputes between aggregators and participants. Recent risk-aware IBDR optimization has considered transaction uncertainty for building users [35], and robust virtual-power-plant-oriented demand response has improved response tracking under uncertainty [36]. These studies indicate that allocation should consider not only expected flexibility, but also reliability and execution risk. However, the credibility of the baseline used for settlement is still not explicitly treated as a pre-event allocation factor or feasibility-related constraint. Therefore, baseline credibility should be considered before an event as a risk-aware resource allocation factor.

Reinforcement learning provides a promising framework for dynamic incentive allocation because it can learn sequential decisions from interactions with changing environments. In incentive-based demand response, an aggregator must adapt incentive levels and response assignments according to renewable generation, net-load conditions, user states, and remaining incentive budgets. Reinforcement learning has been widely studied for sequential decision-making and demand response control [37,38]. Earlier work applied reinforcement learning and deep neural networks to incentive-based demand response, while recent studies have further extended learning-based approaches to integrated demand response, flexible-demand-side microgrid optimization, electric-vehicle charging scheduling under network constraints, cooperative–competitive multi-agent settings, and home energy management under uncertain household parameters [39,40,41,42,43,44]. These studies demonstrate the potential of learning-based methods to adapt demand response decisions to time-varying system and user conditions. For example, integrated IBDR studies have modeled demand-side coupling [40], and multi-agent demand response studies have improved coordination among heterogeneous users [43]. These methods are usually evaluated by operating cost, response tracking, or participation performance.

However, ordinary reinforcement learning is not sufficient for incentive-based demand response because learned actions may violate practical constraints. For example, an unconstrained policy may exceed the incentive budget, assign response tasks beyond user capability, overuse low-reliability users, or rely heavily on users whose baselines are difficult to verify. Constrained reinforcement learning provides a natural way to incorporate such requirements into sequential decision-making, and constrained policy optimization has provided an important theoretical foundation for optimizing rewards under cost constraints [45]. Recent energy-system studies further show that constraint handling is essential when reinforcement learning is applied to building demand response and secure home energy management [46,47]. In power system control, safe reinforcement learning has also been emphasized as a necessary direction because feasibility, reliability, and operational safety are as important as economic performance [48,49]. Robust planning and disturbance-aware control studies in dynamic aerial systems also illustrate the importance of reliable decision-making under time-varying uncertainty [50,51]. Layer-adaptive control has also been used to improve learning efficiency in few-shot tasks, suggesting that adaptive control ideas can complement learning-based decision models [52]. These studies confirm that learned policies require explicit feasibility mechanisms. Nevertheless, the existing constraint sets are mainly concerned with operational safety, comfort, physical limits, or economic budgets, whereas baseline-related settlement credibility is seldom included as a feasibility term before dispatch.

Motivated by these observations, this paper proposes a constrained reinforcement learning method with customer baseline credibility for incentive-based demand response resource allocation. The core idea is to move customer baseline credibility from a post-event settlement reference to a pre-event decision factor. Therefore, the novelty of this study lies in connecting baseline measurement credibility with user resource profiling, constrained group-level learning, and pre-dispatch action correction. The proposed method first evaluates user resource quality by combining flexible capacity, historical response reliability, response cost, and baseline credibility. Users are then grouped into response levels, and a constrained reinforcement learning agent dynamically allocates incentive multipliers and response task ratios among these groups. An action correction mechanism with CBL risk constraints is further designed to check and revise decisions according to budget limits, incentive price bounds, response capacity constraints, and baseline-related risk constraints. Public industrial demand response data and open electricity-system data are used to support baseline calculation, response verification, user reliability evaluation, and renewable energy scenario construction [53,54].

The main contributions of this paper are summarized as follows. First, a demand response resource evaluation framework with customer baseline credibility is proposed. Different from resource evaluation methods that mainly rank users by flexible capacity or historical reliability, the proposed framework incorporates baseline credibility so that demand response resources are evaluated by both adjustability and verifiability. Second, the incentive allocation problem is formulated as a constrained sequential decision-making problem. The proposed method dynamically determines group-level incentive multipliers and response task ratios, which reduces action dimensionality while preserving adaptive decision-making capability. Third, an action correction mechanism with CBL risk constraints is designed to prevent excessive reliance on users with unreliable baselines while maintaining budget feasibility, response capacity feasibility, and settlement credibility. Through this design, incentive-based demand response can be organized not only around how much load can be adjusted, but also around how credibly the adjustment can be measured, verified, and compensated.

## 2. Methodology

### 2.1. Overall Framework

**Figure 1:**
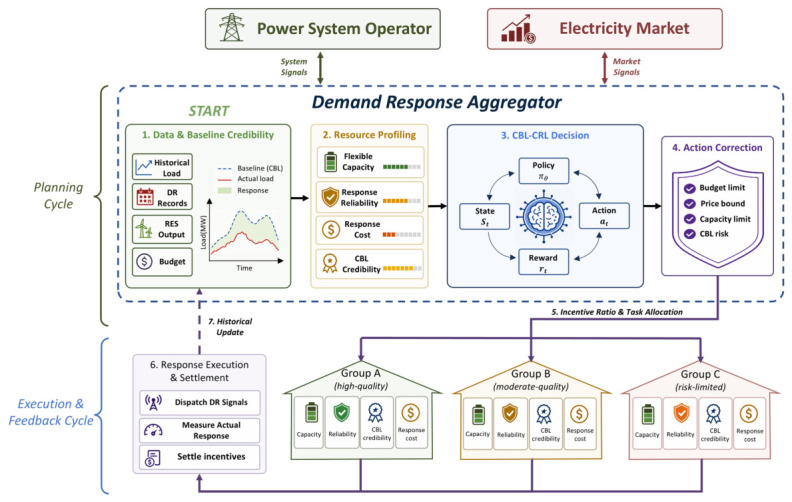
Overall framework of the proposed constrained reinforcement learning method with customer baseline credibility for incentive-based demand response resource allocation. The workflow starts from data inputs and CBL credibility evaluation, and then proceeds through resource profiling, group-level CBL-CRL decision-making, action correction, group response allocation, response execution and settlement, and historical update.

Figure 1 presents the overall framework of the proposed method. The framework is centered on a demand response aggregator that coordinates the power system operator, the electricity market, and heterogeneous demand-side users. The aggregator receives system signals, market signals, user-side historical load records, and demand response event records. These inputs are used for customer baseline credibility evaluation, user resource profiling, and dynamic incentive allocation.

The framework consists of two coupled cycles. In the planning cycle, the aggregator first evaluates customer baseline credibility from historical load and event records. The credibility information is then combined with flexible capacity, response reliability, and response cost to construct user resource profiles. Based on these profiles, users are grouped into different resource levels, which serve as the decision units of the reinforcement learning model.

The CBL-CRL decision module determines group-level incentive multipliers and response task allocation ratios according to the current system state, remaining budget, and group-level resource states. The generated action is further processed by the action correction module, where budget, price, capacity, and CBL risk constraints are checked before implementation. This design avoids direct user-level control and keeps the allocation problem scalable.

In the execution and feedback cycle, the corrected allocation decision is sent to the corresponding user groups. After the demand response event, actual response outcomes and settlement records are collected. These records are used to update user reliability and customer baseline credibility, which then provide updated inputs for subsequent planning cycles.

For clarity, the main symbols used in the proposed framework are summarized before the detailed formulation. Each variable is also defined near its first use in the corresponding subsection.
**Notation summary I: CBL credibility and user profiling**SymbolMeaning*i*, *k*, *t*User, group, and time indices.$CBL_{i}\left(t\right)$Customer baseline load of user *i*.$P_{i}^{\mathrm{act}}\left(t\right)$Actual measured load of user *i*.$\Delta P_{i}\left(t\right)$Measured response of user *i*.$e_{i}^{\mathrm{CBL}}$Normalized CBL error.$q_{i}^{\mathrm{CBL}}$CBL credibility score.ηCredibility sensitivity coefficient.$Cap_{i}$, $Rel_{i}$, $Cost_{i}$User capacity, reliability, and response cost.$S_{i}$User resource quality score.


**Notation summary II: decision model, correction, and metrics**
SymbolMeaning

$X_{k}\left(t\right)$

Group-level resource state.$s_{t}$, $a_{t}$RL state and action.$\kappa_{k}\left(t\right)$, $\rho_{k}\left(t\right)$Incentive multiplier and response allocation ratio.

$\lambda_{k}\left(t\right)$

Incentive price of group *k*.

$\Delta P_{k}^{\mathrm{tar}}\left(t\right)$

Target response assigned to group *k*.

$\Delta P_{\mathrm{DR}}\left(t\right)$

Realized aggregated response.

$\Omega_{t}$

Feasible action set.

$Risk_{\mathrm{CBL}}\left(t\right)$

CBL allocation risk.

$\epsilon_{\mathrm{CBL}}$

CBL risk threshold.$C^{\mathrm{tot}}$, $C_{\mathrm{inc}}$Total operating cost and incentive payment.$\eta_{\mathrm{RE}}$, $E_{\mathrm{track}}$, $E_{\mathrm{CBL}}$Renewable accommodation, tracking error, and CBL risk exposure.

### 2.2. Customer Baseline Load and Credibility Evaluation

Customer baseline load is the reference used to estimate the electricity consumption that a user would have had without a demand response event. In incentive-based demand response, it determines both the measured response quantity and the corresponding incentive payment. Therefore, an inaccurate baseline may lead to over-compensation or under-compensation. To incorporate this measurement issue into resource allocation, this paper introduces customer baseline credibility as a decision-related variable.

For user *i* at time *t*, let $CBL_{i}\left(t\right)$ denote the estimated customer baseline load and $P_{i}^{\mathrm{act}}\left(t\right)$ denote the actual measured load during the demand response event. The measured response quantity is defined as

(1) $$\Delta P_{i}\left(t\right)={\left[CBL_{i}\left(t\right)-P_{i}^{\mathrm{act}}\left(t\right)\right]}_{+},$$

where ${\left[\cdot \right]}_{+}=\max\left\{0,\cdot \right\}$. This operation avoids counting negative response values caused by natural load fluctuations. The aggregated response of a user group can be obtained by summing the measured responses of its users.

The proposed framework does not rely on a specific baseline estimation model. The baseline can be obtained by historical averaging, adjusted historical averaging, regression-based methods, or machine learning-based estimators. This paper focuses on how the quality of the estimated baseline affects resource allocation. Therefore, the baseline estimator is treated as an interchangeable component, while the credibility evaluation is explicitly connected to user profiling and incentive allocation.

To evaluate baseline credibility, non-event validation periods are used. Since no demand response is implemented during these periods, the actual load can be regarded as the reference load for measuring baseline error. The normalized customer baseline error of user *i* is defined as

(2) $$e_{i}^{\mathrm{CBL}}=\frac{\sum_{t\in T_{\mathrm{val}}}\left|CBL_{i}\left(t\right)-P_{i}^{\mathrm{act}}\left(t\right)\right|}{\sum_{t\in T_{\mathrm{val}}}P_{i}^{\mathrm{act}}\left(t\right)},$$

where $T_{\mathrm{val}}$ denotes the validation time set. A smaller value of $e_{i}^{\mathrm{CBL}}$ indicates that the estimated baseline is closer to the actual non-event load and that the response contribution can be measured more reliably.

The customer baseline credibility score is then defined as

(3) $$q_{i}^{\mathrm{CBL}}=\exp\left(-\eta e_{i}^{\mathrm{CBL}}\right),$$

where η>0 is a sensitivity coefficient. The value of $q_{i}^{\mathrm{CBL}}$ lies in (0,1]. The coefficient η has a risk-preference interpretation in the proposed framework. It controls how strongly the normalized baseline error is converted into a credibility penalty. When η is small, the credibility score decreases slowly as the baseline error increases, meaning that the aggregator adopts a less conservative attitude toward moderate baseline errors. When η is large, the same baseline error leads to a much lower credibility score, meaning that the aggregator becomes more risk-averse to baseline uncertainty and settlement error. Therefore, η can be regarded as a tunable parameter reflecting the aggregator’s tolerance for baseline-related settlement risk. A higher value indicates higher baseline credibility and lower settlement risk. This score is used in the following resource profiling and action correction steps, allowing the allocation process to account for baseline-related settlement uncertainty.

### 2.3. User Resource Profiling and Grouping

After customer baseline credibility is obtained, heterogeneous users are converted into structured demand response resources. In incentive-based demand response, users differ in flexible capacity, response stability, compensation requirement, and response measurement credibility. User heterogeneity has been widely observed in behavioral demand response, building-level controllability, appliance-level flexibility, and data-driven demand response potential modeling [24,25,26,27].

Direct user-level incentive allocation makes the action space grow with the number of enrolled users. It also requires the policy to frequently adjust individual user selections and response shares as user states change. Reinforcement learning-based demand response studies therefore require scalable state–action representations and coordinated allocation mechanisms [38,43]. For this reason, users are first profiled and grouped before reinforcement learning-based allocation.

The user resource profile contains four attributes: flexible capacity, response reliability, response cost, and customer baseline credibility. Flexible capacity represents the load adjustment potential under current operating conditions. Response reliability reflects historical execution performance. Response cost measures the compensation requirement or operational burden associated with load adjustment. Customer baseline credibility represents the reliability of response measurement and settlement. These attributes define the dispatch value of a user from both operational and settlement perspectives.

Let $Cap_{i}$, $Rel_{i}$, $Cost_{i}$, and $q_{i}^{\mathrm{CBL}}$ denote the normalized flexible capacity, response reliability, response cost, and customer baseline credibility of user *i*, respectively. The resource quality score of user *i* is defined as

(4) $$S_{i}=w_{\mathrm{cap}}Cap_{i}+w_{\mathrm{rel}}Rel_{i}+w_{\mathrm{cbl}}q_{i}^{\mathrm{CBL}}-w_{\mathrm{cost}}Cost_{i},$$

where $w_{\mathrm{cap}}$, $w_{\mathrm{rel}}$, $w_{\mathrm{cbl}}$, and $w_{\mathrm{cost}}$ are non-negative weights. The positive terms represent desirable demand response properties, while the cost term reduces the resource score. When response verification and settlement fairness are emphasized, a larger weight can be assigned to customer baseline credibility.

The default setting uses three groups because it provides an interpretable high–medium–risk-limited resource structure for allocation. Group A represents priority response resources, group B represents regular response resources, and group C represents conservative response resources. This setting keeps the group-level action space compact while preserving the main operational differences among users. The grouping mechanism itself is not restricted to three groups; when finer resource granularity is required, the same profiling rule can be extended to *K* groups by sorting users according to $S_{i}$ and applying percentile-based thresholds.

The implementation settings of the profiling and grouping process are summarized in Table 1.

The three groups are allocation-oriented resource classes. Group A is used as the priority response group, group B as the regular response group, and group C as the conservative response group. This structure avoids direct user-level control because the reinforcement learning agent allocates incentive multipliers and response task ratios to groups. With three groups, the action contains three incentive multipliers and three response allocation ratios.

For each group k∈{A,B,C}, the aggregator constructs a group-level state vector:

(5) $$X_{k}\left(t\right)=\left[Cap_{k}\left(t\right),Rel_{k}\left(t\right),Cost_{k}\left(t\right),{\bar{q}}_{k}^{\mathrm{CBL}}\left(t\right)\right],$$

where $Cap_{k}\left(t\right)$, $Rel_{k}\left(t\right)$, $Cost_{k}\left(t\right)$, and ${\bar{q}}_{k}^{\mathrm{CBL}}\left(t\right)$ denote group-level flexible capacity, response reliability, response cost, and average customer baseline credibility, respectively. These group-level states are the decision inputs for resource allocation. The online action dimension is independent of the number of enrolled users, while user differences are retained through group-level capacity, reliability, cost, and CBL credibility features.

The grouping result is updated when new demand response records become available. Users with stable response completion and credible baselines may receive higher resource scores, whereas users with unstable responses or large baseline errors may be assigned to more conservative roles in later events. Therefore, the profiling and grouping mechanism is updated by response history and customer baseline credibility.

### 2.4. Constrained Reinforcement Learning Decision Model

After user resource grouping, the incentive allocation problem is formulated as a group-level sequential decision-making problem. At each decision period, the demand response aggregator observes the current system condition and the resource states of different user groups, and then determines the incentive intensity and response task share for each group. Compared with direct user-level control, group-level decision-making reduces the action dimension and makes the allocation policy easier to implement in practical demand response programs.

The group-level formulation is adopted for both scalability and implementation reasons. If the aggregator directly optimized user-level incentive multipliers and response ratios for all enrolled users, the policy would have to output two decision variables for each user. In contrast, the proposed framework first maps heterogeneous users into a small number of resource groups and then lets the policy decide group-level incentive multipliers and response shares.

For a general setting with *K* resource groups, the policy outputs 2K continuous decision variables, including *K* incentive multipliers and *K* response allocation ratios. In the default implementation, K=3, so the policy outputs six continuous decision variables. This keeps the action dimension compact and independent of the number of enrolled users. User-level heterogeneity is not ignored, because flexible capacity, response reliability, response cost, and CBL credibility are first embedded into the resource quality score and then aggregated into group-level state features. This design also matches aggregator operation, where users are commonly managed as portfolios or resource classes.

The state at time *t* is defined as

(6) $$s_{t}=\left[P_{\mathrm{net}}\left(t\right),P_{\mathrm{RE}}\left(t\right),\Delta P^{\mathrm{tar}}\left(t\right),B_{\mathrm{rem}}\left(t\right),X_{A}\left(t\right),X_{B}\left(t\right),X_{C}\left(t\right)\right],$$

where $P_{\mathrm{net}}\left(t\right)$ is the system net load, $P_{\mathrm{RE}}\left(t\right)$ is the available renewable generation, $\Delta P^{\mathrm{tar}}\left(t\right)$ is the target response requirement, $B_{\mathrm{rem}}\left(t\right)$ is the remaining incentive budget, and $X_{A}\left(t\right)$, $X_{B}\left(t\right)$, and $X_{C}\left(t\right)$ are the group-level resource states. This state representation allows the agent to consider both system-side requirements and demand-side resource quality.

The raw action generated by the agent is defined as

(7) $$a_{t}=\left[\kappa_{A}\left(t\right),\kappa_{B}\left(t\right),\kappa_{C}\left(t\right),\rho_{A}\left(t\right),\rho_{B}\left(t\right),\rho_{C}\left(t\right)\right],$$

where $\kappa_{k}\left(t\right)$ is the incentive multiplier for group *k*, and $\rho_{k}\left(t\right)$ is the response task allocation ratio assigned to group *k*, with k∈{A,B,C}. The raw allocation ratios satisfy $\sum_{k}\rho_{k}\left(t\right)=1$ and $\rho_{k}\left(t\right)\geq 0$. Thus, the agent does not assign incentives to each individual user, but allocates incentive intensity and response responsibility among resource groups.

Given the action, the incentive price and target response task of group *k* are calculated as

(8) $$\lambda_{k}\left(t\right)=\lambda_{0}\left(t\right)\kappa_{k}\left(t\right),\;\Delta P_{k}^{\mathrm{tar}}\left(t\right)=\rho_{k}\left(t\right)\Delta P^{\mathrm{tar}}\left(t\right),$$

where $\lambda_{0}\left(t\right)$ is the base incentive price. This formulation connects the system-level response requirement with group-level allocation decisions. A larger incentive multiplier increases the compensation level of a group, while a larger allocation ratio assigns a greater share of the response target to that group.

The reward function is designed to reflect economic efficiency and system operation performance:

(9) $$r_{t}=-\alpha_{1}C_{\mathrm{sys}}\left(t\right)-\alpha_{2}C_{\mathrm{inc}}\left(t\right)-\alpha_{3}\left|\Delta P_{\mathrm{DR}}\left(t\right)-\Delta P^{\mathrm{tar}}\left(t\right)\right|-\alpha_{4}P_{\mathrm{curt}}\left(t\right),$$

where $C_{\mathrm{sys}}\left(t\right)$ is the system operating cost, $C_{\mathrm{inc}}\left(t\right)$ is the incentive payment, $\Delta P_{\mathrm{DR}}\left(t\right)$ is the realized aggregated response, and $P_{\mathrm{curt}}\left(t\right)$ is the renewable curtailment. The four terms penalize operating cost, incentive expenditure, response tracking error, and renewable curtailment, respectively.

The decision model is formulated as a constrained reinforcement learning problem:

(10) $$\max_{\pi_{\theta }}E_{\pi_{\theta }}\left[\sum_{t=1}^{T}\gamma^{t-1}r_{t}\right],\;s.t.\;E_{\pi_{\theta }}\left[\sum_{t=1}^{T}g_{m}\left(s_{t},a_{t}\right)\right]\leq d_{m},\;m=1,\ldots ,M,$$

where $\pi_{\theta }$ is the parameterized policy, γ is the discount factor, $g_{m}\left(s_{t},a_{t}\right)$ is the *m*-th constraint cost, and $d_{m}$ is the corresponding threshold. The detailed implementation of the budget, price, capacity, and CBL risk constraints is handled by the action correction module in the next subsection.

### 2.5. Action Correction

The raw action generated by the CBL-CRL decision module may not always satisfy the implementation requirements of an incentive-based demand response event. Therefore, an action correction module is introduced to convert the raw action into a feasible allocation decision before execution. Let $a_{t}^{\mathrm{RL}}$ be the raw action at time *t*, and let $a_{t}^{\mathrm{cor}}$ be the corrected action. The correction is formulated as

(11) $$a_{t}^{\mathrm{cor}}=\arg\min_{a\in \Omega_{t}}{∥a-a_{t}^{\mathrm{RL}}∥}_{2}^{2},$$

where $\Omega_{t}$ denotes the feasible action set.

The feasible set is defined as

(12) $$\begin{array}{cc} \Omega_{t}=\{a:\; & C_{\mathrm{inc}}\left(t\right)\leq B_{\mathrm{rem}}\left(t\right),\\ \; & \lambda_{\min}\leq \lambda_{k}\left(t\right)\leq \lambda_{\max},\\ \; & 0\leq \rho_{k}\left(t\right)\leq 1,\;\sum_{k\in \{A,B,C\}}\rho_{k}\left(t\right)\leq 1,\\ \; & 0\leq \Delta P_{k}^{\mathrm{tar}}\left(t\right)\leq Cap_{k}\left(t\right),\\ \; & Risk_{\mathrm{CBL}}\left(t\right)\leq \epsilon_{\mathrm{CBL}},\;k\in \left\{A,B,C\right\}\}. \end{array}$$

These constraints respectively regulate incentive expenditure, price feasibility, allocation-ratio feasibility, group response capacity, and baseline-related allocation risk. The raw allocation ratios generated by the policy are normalized to sum to one before correction. After correction, the dispatched allocation ratios are allowed to sum to less than one when the full target response cannot be assigned without violating budget, capacity, or CBL risk constraints. The unassigned response requirement is then reflected through the response tracking term in the reward function.

The incentive cost and CBL risk are calculated as

(13) $$C_{\mathrm{inc}}\left(t\right)=\sum_{k\in \{A,B,C\}}\lambda_{k}\left(t\right)\Delta P_{k}^{\mathrm{tar}}\left(t\right),\;Risk_{\mathrm{CBL}}\left(t\right)=\sum_{k\in \{A,B,C\}}\rho_{k}\left(t\right)\left(1-{\bar{q}}_{k}^{\mathrm{CBL}}\left(t\right)\right).$$

The CBL risk term increases when more response tasks are assigned to groups with lower average baseline credibility. In implementation, the projection can be approximated by a sequential correction procedure: incentive multipliers are clipped, allocation ratios are normalized or reduced, and response tasks are reduced or reallocated when budget, capacity, or CBL risk limits are violated.

The feasibility boundary of the correction problem is determined by the active constraints in the feasible set. The budget boundary is reached when the incentive cost equals the remaining budget. The price boundary is reached when an incentive price reaches its lower or upper bound. The capacity boundary is reached when the assigned response task of a group equals its available flexible capacity. The CBL risk boundary is reached when the weighted allocation risk equals the prescribed CBL risk threshold.

The feasible set is non-empty under mild implementation conditions. Specifically, the price interval must be non-empty, the remaining budget and group capacities must be non-negative, and the CBL risk threshold must be non-negative. Under these conditions, a conservative zero-response allocation is feasible because it produces zero incentive cost, zero assigned response capacity, and zero CBL allocation risk. Therefore, even when the full response target cannot be feasibly assigned, the correction module can still return an executable reduced-response decision.

The existence of the corrected action also follows from the structure of the projection problem. The feasible set is closed and bounded because incentive prices and allocation ratios are bounded, and budget, capacity, and CBL risk are defined by closed inequality constraints. The squared-distance objective is continuous with respect to the action variables. Therefore, when the feasible set is non-empty, at least one minimizer exists for the action correction problem.

For the constrained reinforcement learning formulation, the finite-horizon decision process has bounded rewards and bounded constraint costs because incentive prices, allocation ratios, response capacities, and budgets are bounded by construction. Since the conservative feasible allocation described above provides at least one feasible policy, the feasible policy set is non-empty. The actor–critic training procedure is then used to approximate a high-quality feasible policy, while the action correction module guarantees that the action dispatched at each event satisfies the defined feasibility constraints.

### 2.6. Response Execution, Settlement, and Historical Update

After action correction, the feasible allocation decision is dispatched to the corresponding user groups. The decision includes the incentive multiplier and the response task allocation ratio of each group. During the demand response event, users adjust their loads according to the received incentive signal, their available flexible capacity, and their own operating conditions. After the event, the aggregator measures the actual load and calculates the delivered response based on the customer baseline load defined in Section 2.2.

For group *k*, the realized group response is obtained by aggregating the measured responses of all users in the group

(14) $$\Delta P_{k}^{act}\left(t\right)=\sum_{i\in G_{k}}\Delta P_{i}\left(t\right),\;\Delta P_{DR}\left(t\right)=\sum_{k\in \{A,B,C\}}\Delta P_{k}^{act}\left(t\right).$$

Here, $\Delta P_{i}\left(t\right)$ is the measured response of user *i* calculated from the customer baseline and the actual event-period load. The aggregated response $\Delta P_{DR}\left(t\right)$ is used to evaluate whether the demand response target is achieved and is also used in the reward function of the decision model.

The incentive settlement is based on the measured response quantity. For user *i*, the settlement payment is calculated as

(15) $$R_{i}=\sum_{t\in T_{DR}}\lambda_{c(i)}\left(t\right)\Delta P_{i}\left(t\right),$$

where $T_{DR}$ is the demand response event period, c(i) denotes the group of user *i*, and $\lambda_{c(i)}\left(t\right)$ is the incentive price of the corresponding group. This settlement rule links the user’s compensation to its measured contribution and keeps the payment mechanism consistent with customer baseline verification.

The response outcome is then used to update user reliability. Specifically, the reliability of a user is updated according to the ratio between its measured response and assigned response target, using a moving-average update rule. Users that repeatedly complete their assigned response tasks obtain higher reliability scores, whereas users with frequent under-response receive lower reliability scores in subsequent resource profiling.

Customer baseline credibility is also updated when new baseline error information becomes available. Let $e_{i}^{CBL,new}\left(t\right)$ be the newly observed baseline error after the event or validation period. The credibility score is updated as

(16) $$q_{i}^{CBL}\left(t+1\right)=\left(1-\nu \right)q_{i}^{CBL}\left(t\right)+\nu \exp\left(-\eta e_{i}^{CBL,new}\left(t\right)\right),$$

where ν∈(0,1) is the update coefficient. This update allows the framework to adjust the credibility of each user according to newly observed measurement performance.

Through the above update mechanism, the proposed method forms a closed-loop resource allocation process. The execution result of each demand response event affects subsequent user profiling, group assignment, and incentive allocation. Users with stable response performance and credible baselines may receive higher priority in future events, whereas users with unstable responses or unreliable baselines are gradually assigned to more conservative roles.

The complete procedure of the proposed CBL-CRL method is summarized in Algorithm 1.
**Algorithm 1** CBL-CRL Resource Allocation.  1:Initialize user profiles $\left\{Cap_{i},Rel_{i},Cost_{i},q_{i}^{CBL}\right\}$.  2:**for** each DR event **do**  3:      Estimate $CBL_{i}$ and compute $q_{i}^{CBL}$ for all users.  4:      Build user profiles and form groups $G_{A}$, $G_{B}$, and $G_{C}$.  5:      **for** each period *t* **do**  6:            Observe state $s_{t}$ and generate raw action $a_{t}^{RL}=\pi_{\theta }\left(s_{t}\right)$.  7:            Correct $a_{t}^{RL}$ under budget, price, capacity, and CBL risk constraints.  8:            Execute $a_{t}^{cor}$, measure response, and settle incentives.  9:            Store response and settlement records.10:      **end for**11:      Update user profiles; update policy parameters during training.12:**end for**

## 3. Case Study and Experimental Setup

### 3.1. Data Preparation and Scenario Construction

The user-side data are taken from the public dataset of South Korean manufacturing factories participating in demand response programs [53]. The dataset contains 10 factory-level load files, each recording one-minute electricity consumption from 1 March to 30 September 2019. Each factory file contains 308,160 time-stamped load records, corresponding to 214 days of measurements. In total, the raw user-side dataset contains approximately 3.08 million one-minute load observations. In addition to load records, the dataset provides manufacturing-type information, demand response participation records, mandatory reduction capacities, and actual response capacities. These fields are used to calibrate user-side flexible capacity, response reliability, customer baseline error, and CBL credibility.

The experiments are conducted at an hourly resolution. The one-minute load records are aggregated into hourly average load values, resulting in 5136 hourly samples for each factory before filtering. An hourly sample is regarded as valid when at least 80% of its corresponding one-minute measurements are available. Short missing intervals are filled by linear interpolation before hourly aggregation. Days with more than 5% invalid hourly samples are excluded from the baseline reference pool. For each factory, extreme hourly values are screened using a three-standard-deviation rule and are replaced by the nearest valid bound. Non-event days are used for customer baseline construction and validation, while recorded demand response event days are used to calculate realized response and response reliability.

For customer baseline calculation, a similar-day historical reference rule is adopted. For each event hour, valid non-event days with the same hour-of-day index are selected as reference samples, excluding demand response event days and low-quality days. The rolling non-event validation samples are used to compute the normalized CBL error and CBL credibility defined in Section 2.2. This setting keeps the baseline calculation auditable and avoids making the case study dependent on a particular black-box baseline estimator.

Because the public dataset contains only 10 factories, it is used as the empirical basis for constructing an expanded user pool. In the base case, 50 demand response users are generated from the 10 factory prototypes. For each generated user, one factory prototype is randomly selected, and its normalized load profile is scaled by a factor sampled from [0.7,1.3]. The response capacity is perturbed within ±15% of the prototype capacity ratio. User reliability and CBL error are sampled from the empirical distributions obtained from the real records. Since monetary response cost is not directly reported in the dataset, a normalized response cost attribute is generated within a bounded range and kept identical across all compared methods. This expanded user pool preserves the statistical characteristics of the industrial data while providing enough users and episodes for reinforcement learning training.

The system-side data are constructed from the Open Power System Data time-series package [54]. Hourly system load, wind generation, solar generation, and day-ahead price data from 2019 are used. Wind and solar generation are aggregated as available renewable generation. Because the user-side industrial records and the system-side electricity time series are collected from different public datasets, the system-side time series are normalized and scaled to the aggregate size of the constructed user pool. This scaling procedure is used to align the magnitude of system-side net-load stress with the available demand-side flexibility and to provide a consistent evaluation environment for all compared methods. The same scaled system data, user pool, event-triggering rule, and budget setting are used throughout the experiments.

Demand response events are triggered by net-load stress. The net load and the target response requirement are defined as

(17) $$P_{\mathrm{net}}\left(t\right)=P_{\mathrm{load}}\left(t\right)-P_{\mathrm{RE}}\left(t\right),\;\Delta P^{\mathrm{tar}}\left(t\right)=\min\left\{{\left[P_{\mathrm{net}}\left(t\right)-P_{\mathrm{net}}^{\mathrm{thr}}\right]}_{+},Cap_{\mathrm{tot}}\left(t\right)\right\},$$

where $P_{\mathrm{load}}\left(t\right)$ is the system load, $P_{\mathrm{RE}}\left(t\right)$ is the available renewable generation, $P_{\mathrm{net}}^{\mathrm{thr}}$ is the net-load triggering threshold, and $Cap_{\mathrm{tot}}\left(t\right)$ is the total available flexible capacity of the user pool. In the experiments, $P_{\mathrm{net}}^{\mathrm{thr}}$ is set as the 90th percentile of the training-period net-load series. This rule ties the response target to system operating stress and prevents the assigned response requirement from exceeding available demand-side flexibility. It also reduces subjective event selection by generating demand response tasks from a fixed percentile-based net-load criterion.

Four operating scenarios are constructed for evaluation. The normal scenario uses the original scaled system data and user parameters. In the high-renewable scenario, wind and solar generation are increased by 30% to examine allocation performance under stronger renewable variability. In the peak-load scenario, system load is increased by 15% to create higher net-load stress. In the high-uncertainty scenario, user response deviation is increased by 50%, and user reliability is reduced by 10% to test robustness under stronger behavioral uncertainty. These scenarios are used as controlled stress cases for comparative evaluation. They vary one dominant factor at a time, including renewable generation, load stress, and user-side response uncertainty, so that the influence of each factor on the allocation method can be examined more clearly.

### 3.2. Evaluation Metrics

The performance of each method is evaluated from three aspects: system operation, incentive efficiency, and baseline-related settlement risk. All metrics are calculated using the same user pool, event-triggering rule, budget level, and scenario data. Unless otherwise specified, the metrics are computed based on the actual response measured after execution.

The total operating cost is defined as the sum of the system operating cost, the incentive payment, and a curtailment-related penalty:

(18) $$C^{tot}=\sum_{t}\left(C_{sys}\left(t\right)+C_{inc}\left(t\right)+c_{curt}P_{curt}\left(t\right)\right),\;C_{inc}\left(t\right)=\sum_{k\in \{A,B,C\}}\lambda_{k}\left(t\right)\Delta P_{k}^{act}\left(t\right),$$

where $C_{sys}\left(t\right)$ is the system operating cost, $C_{inc}\left(t\right)$ is the actual incentive payment, $P_{curt}\left(t\right)$ is the system-level renewable curtailment indicator, and $c_{curt}$ is the curtailment penalty coefficient. In this study, demand response events are triggered by high net-load stress. Therefore, $P_{curt}\left(t\right)$ and the corresponding renewable accommodation rate are used as auxiliary system-level indicators.

Renewable accommodation and response tracking error are evaluated by

(19) $$\eta_{\mathrm{RE}}=1-\frac{\sum_{t}P_{\mathrm{curt}}\left(t\right)}{\sum_{t}P_{\mathrm{RE}}\left(t\right)},\;E_{\mathrm{track}}=\frac{\sum_{t}\left|\Delta P_{\mathrm{DR}}\left(t\right)-\Delta P^{\mathrm{tar}}\left(t\right)\right|}{\sum_{t}\Delta P^{\mathrm{tar}}\left(t\right)+\epsilon },$$

where $\eta_{\mathrm{RE}}$ is the renewable accommodation rate, $E_{\mathrm{track}}$ is the normalized response tracking error, and ϵ is a small positive constant used to avoid division by zero. The renewable accommodation rate measures the proportion of available renewable generation that is not curtailed in the tested system setting. A higher $\eta_{\mathrm{RE}}$ therefore indicates better renewable utilization and lower curtailment-related penalty. In this study, demand response events are triggered by high net-load stress, so $\eta_{\mathrm{RE}}$ is used as an auxiliary system-level indicator.

The response tracking error measures how closely the realized aggregated response follows the target response requirement. The numerator accumulates the absolute deviation between the realized response $\Delta P_{\mathrm{DR}}\left(t\right)$ and the target response $\Delta P^{\mathrm{tar}}\left(t\right)$, while the denominator normalizes this deviation by the total target response. A lower $E_{\mathrm{track}}$ means that the dispatched demand response resources follow the target response more accurately. Therefore, $E_{\mathrm{track}}$ is reported as an error metric, and lower values indicate better response tracking performance.

To evaluate whether incentive payments are used efficiently, the incentive efficiency is defined as

(20) $$\eta_{inc}=\frac{C_{NoDR}^{tot}-C_{method}^{tot}}{\sum_{t}C_{inc}\left(t\right)+\varepsilon },$$

where $C_{NoDR}^{tot}$ is the total operating cost in the no-demand-response reference case, and $C_{method}^{tot}$ is the total operating cost under the evaluated method. This metric measures the system cost reduction obtained per unit of incentive payment.

The baseline-related allocation risk is measured by CBL risk exposure:

(21) $$E_{CBL}=\frac{\sum_{t}\sum_{k\in \{A,B,C\}}\left(1-{\bar{q}}_{k}^{CBL}\left(t\right)\right)\Delta P_{k}^{tar}\left(t\right)}{\sum_{t}\sum_{k\in \{A,B,C\}}\Delta P_{k}^{tar}\left(t\right)+\varepsilon }.$$

A lower $E_{CBL}$ indicates that fewer response tasks are assigned to user groups with low baseline credibility. This metric is used to examine whether the proposed method improves not only system-level performance, but also the credibility of response allocation and settlement.

### 3.3. Implementation Settings

All experiments are implemented in Python 3.11. The demand response environment is constructed at an hourly resolution, and one episode corresponds to one operating day with 24 decision periods. In periods where the net-load threshold is not exceeded, the target response is set to zero and the no-response task is dispatched. When a demand response event is triggered, the trained policy generates group-level incentive multipliers and response allocation ratios, followed by the action correction procedure described in Section 2.5.

The dataset is split chronologically into training, validation, and testing subsets. The first 70% of the available days are used for training, the next 15% are used for validation, and the remaining 15% are used for testing. All load, renewable generation, budget, capacity, cost, reliability, and CBL credibility variables are normalized using statistics computed from the training subset. The same normalization parameters are then applied to the validation and testing subsets to avoid information leakage.

The CBL-CRL policy is implemented using an actor–critic neural network. Both the actor and critic networks use two fully connected hidden layers with 128 neurons and ReLU activation. The policy output contains two parts. The first part generates the incentive multipliers for the three user groups, which are mapped to the range [0.6,1.5]. The second part generates the response allocation logits, which are converted into allocation ratios by a softmax operation. The allocation ratios are then passed to the action correction module together with the incentive multipliers.

PPO and PPO-Penalty are trained using a PPO-style actor–critic update. For PPO-Penalty, budget, price, capacity, and CBL risk violations are added to the reward function as penalty terms. SAC is trained using the soft actor–critic update. MAPPO follows a centralized-training decentralized-execution implementation, where the three user groups are treated as cooperative agents. CBL-CRL uses the same actor–critic backbone as PPO, followed by the action correction module described in Section 2.5. The learning rate is set to $3\times 10^{-4}$, the discount factor is set to 0.99, the clipping parameter is set to 0.2, and the mini-batch size is set to 256. The policy is trained for 300,000 environment steps in the base case. The best policy is selected according to validation performance, considering both total operating cost and CBL risk exposure. During testing, the policy is evaluated without exploration noise. To evaluate computational time, two indicators are added. $T_{\mathrm{train}}$ is measured as the wall-clock time required to complete 300,000 environment steps. $T_{\mathrm{dec}}$ is measured as the average online decision time per decision period on the test set. For CBL-CRL, $T_{\mathrm{dec}}$ includes state processing, policy inference, and action correction. Rule-based methods have no offline training time.

The initial daily incentive budget is set to 80% of the payment that would be required if all triggered response targets were compensated at the base incentive price. This setting creates a budget-constrained allocation problem. The same budget level is used for all compared methods and all ablation variants. For robustness, each experiment is repeated with five random seeds, and the reported results are averaged over these runs.

The key hyperparameters used in the experiments are summarized in Table 2.

## 4. Results and Discussion

### 4.1. Overall Performance and Representative Operation Behavior

This subsection evaluates the proposed CBL-CRL method against representative rule-based and learning-based allocation methods under the normal scenario. The baseline algorithms are selected to cover capacity-based allocation, resource-quality-based allocation, standard on-policy reinforcement learning, reward-penalty-based constraint handling, off-policy continuous control, and multi-agent coordination. This setting allows the proposed method to be compared with both practical rule-based strategies and representative learning-based allocation strategies under the same user pool, event-triggering rule, budget level, and scenario data.

The reference case is No-DR, where no demand response incentive is activated and user loads remain unchanged during response periods. The rule-based strategies include UNI-Cap and SHI-Fixed. UNI-Cap applies a fixed uniform incentive price and allocates response tasks in proportion to available flexible capacity, while SHI-Fixed first groups users according to the resource quality score and then applies fixed group-level incentive multipliers, with $\kappa_{A}=1.2$, $\kappa_{B}=1.0$, and $\kappa_{C}=0.85$. These two rule-based baselines examine whether simple capacity-proportional allocation or fixed resource-quality-based grouping is sufficient for the allocation task.

The learning-based baselines include PPO, PPO-Penalty, SAC, and MAPPO. PPO uses the same group-level state and action structure as CBL-CRL, but only applies basic action clipping without the proposed action correction module [55]. PPO-Penalty adopts the same PPO policy structure, but adds penalty terms for budget, price, capacity, and CBL risk violations to the reward function. SAC uses the soft actor–critic algorithm as an off-policy continuous-control baseline [56], while MAPPO treats the three user groups as cooperative agents and applies a centralized-training decentralized-execution structure [57]. These learning-based baselines evaluate whether CBL-CRL provides additional benefits beyond standard policy learning, soft penalty-based constraint handling, off-policy learning, and cooperative multi-agent allocation.

In addition to performance metrics, Table 3 reports the computational time of each method. The rule-based methods have no offline training stage and require only 0.04–0.06 ms for online decision-making. Among the learning-based methods, PPO has the shortest training time, while MAPPO requires the longest training time due to its multi-agent centralized-training structure. CBL-CRL requires 10.4 min for offline training, which is higher than PPO and PPO-Penalty but lower than SAC and MAPPO. Its online decision time is 0.33 ms, including policy inference and action correction. This indicates that the additional correction step introduces limited online latency.

The online computational complexity of CBL-CRL mainly comes from group-level state construction, actor-network inference, and action correction. With *K* resource groups, the action dimension is 2K, including *K* incentive multipliers and *K* response allocation ratios. The sequential action correction checks price, budget, capacity, and CBL-risk constraints over *K* groups, resulting in O(K) correction complexity. User profiling and percentile-based grouping require O(NlogN) complexity over *N* users and can be updated before dispatch when new records are available. In this study, *K* is set to 3, so the online action dimension remains six.

As shown in Table 3, all demand-response-based methods reduce the normalized total operating cost compared with the No-DR reference case. UNI-Cap reduces $C^{\mathrm{tot}}$ from 1.000 to 0.946±0.007, while SHI-Fixed further reduces it to 0.929±0.006, indicating that activating demand-side flexibility is beneficial and that differentiating users by resource quality is more effective than applying a uniform incentive to all users. The learning-based methods further improve the operational performance. PPO, SAC, and MAPPO reduce $C^{\mathrm{tot}}$ to 0.918±0.012, 0.912±0.008, and 0.905±0.009, respectively, and their response tracking errors decrease from 0.157±0.012 for PPO to 0.121±0.011 for MAPPO. PPO-Penalty provides an additional comparison for reward-penalty-based constraint handling. Compared with PPO, it reduces $E_{\mathrm{CBL}}$ from 0.166±0.021 to 0.132±0.016, showing that CBL-related penalty terms can mitigate baseline-related allocation risk. However, this improvement is accompanied by a higher incentive payment of 0.052 and a lower incentive efficiency of 1.62, and its response tracking error remains higher than those of SAC, MAPPO, and CBL-CRL. This suggests that reward penalties can reduce CBL risk to some extent, but they do not fully replace explicit feasibility correction.

The proposed CBL-CRL method achieves the lowest normalized total operating cost and the highest incentive efficiency among all evaluated methods. It reduces $C^{\mathrm{tot}}$ to 0.897±0.006 and improves $\eta_{\mathrm{inc}}$ to 2.10, corresponding to a 10.3% reduction in normalized total cost compared with No-DR. MAPPO uses a slightly lower incentive payment of 0.047, while CBL-CRL uses 0.049. Nevertheless, CBL-CRL obtains a larger system-level cost reduction and therefore achieves higher incentive efficiency. Its advantage is particularly evident in response tracking and CBL risk control. Compared with MAPPO, CBL-CRL reduces $E_{\mathrm{track}}$ from 0.121±0.011 to 0.108±0.010, while reducing $E_{\mathrm{CBL}}$ from 0.147±0.019 to 0.087±0.009. In relative terms, CBL-CRL reduces the response tracking error by 10.7 percent and the CBL risk exposure by 40.8 percent compared with MAPPO. The normalized total operating cost is also reduced from 0.905 to 0.897, corresponding to a 0.9 percent reduction, while the renewable accommodation rate remains unchanged at 0.970. Compared with PPO-Penalty, CBL-CRL further reduces $E_{\mathrm{CBL}}$ by 0.045 and improves the tracking error by 0.042. Relative to PPO-Penalty, the reductions are 28.0 percent in response tracking error and 34.1 percent in CBL risk exposure. Relative to SHI-Fixed, the corresponding reductions are 47.8 percent and 40.0 percent. These quantitative differences show that the proposed method provides additional benefits beyond fixed group-level incentive allocation, reward-penalty-based constraint handling, and multi-agent coordination. The renewable accommodation rate reaches 0.970 for both MAPPO and CBL-CRL. Since the demand response events in this study are triggered by high net-load stress, renewable accommodation is interpreted as an auxiliary curtailment-related indicator. The result shows that CBL-CRL does not degrade renewable-curtailment-related performance while improving response tracking and substantially reducing baseline-related settlement risk. Therefore, the main advantage of CBL-CRL is a more balanced performance among system operation, response tracking, action feasibility, and baseline-related settlement reliability.

**Figure 2:**
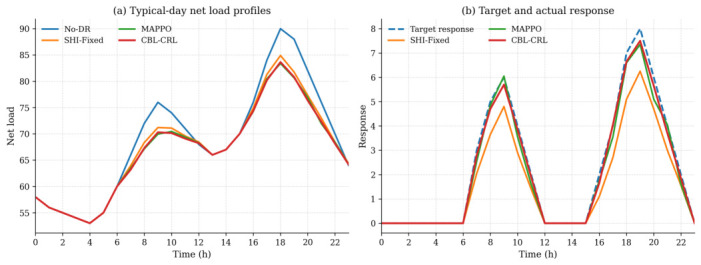
Operational behavior of representative methods under a typical day. (**a**) Typical-day net-load profiles. (**b**) Target and actual response during the demand response periods.

While Table 3 summarizes the overall test-set performance, Figure 2 further illustrates how the performance differences appear in a representative operating day with two demand response events. The comparison includes No-DR, SHI-Fixed, MAPPO, and CBL-CRL, which represent the no-response reference case, the rule-based benchmark, the strongest learning-based baseline, and the proposed method, respectively. As shown in Figure 2a, all demand-response-based methods reduce the net load during event periods compared with No-DR. SHI-Fixed provides a certain level of peak reduction, but its post-response net load remains higher than those of MAPPO and CBL-CRL during the main response windows, showing the limited adaptability of fixed group-level incentives. MAPPO and CBL-CRL show stronger peak-reduction behavior, especially during the evening event. MAPPO is slightly more aggressive in several peak hours, leading to lower net load in some periods, whereas CBL-CRL provides a more balanced response profile at every hour. A small rebound is also observed after the response windows, reflecting post-event load recovery.

Figure 2b compares the target response and the actual realized response. SHI-Fixed shows a clear under-response pattern in both response windows, and the gap becomes larger when the target response increases. MAPPO tracks the target more closely than SHI-Fixed, confirming the advantage of adaptive learning-based allocation over fixed incentive rules. CBL-CRL generally follows the target trajectory closely, while MAPPO shows comparable or slightly more aggressive tracking in several event periods. This behavior is consistent with the results in Table 3: CBL-CRL balances response tracking and baseline-related allocation risk in the allocation process. Overall, Table 3 and Figure 2 together show that the benefit of CBL-CRL comes from combining adaptive group-level allocation with customer baseline credibility. Group-level learning improves the adaptability of incentive allocation compared with fixed rule-based strategies, while customer baseline credibility and action correction reduce baseline-related allocation risk and improve settlement reliability.

### 4.2. Learning Behavior and Constraint Feasibility

**Figure 3:**
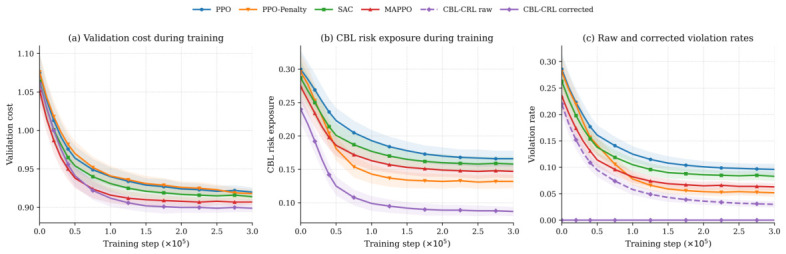
Learning behavior and constraint feasibility of representative learning-based methods. The curves report the mean values over five random seeds, and shaded bands indicate one standard deviation. (**a**) Validation cost during training. (**b**) CBL risk exposure during training. (**c**) Raw and corrected violation rates during training. The labels CBL-CRL raw and CBL-CRL corrected denote the raw policy output before action correction and the executable action after correction, respectively.

Table 3 reports the final test-set performance, and Figure 2 illustrates representative operating behavior on a typical day. Figure 3 complements these results by showing how the learning-based methods evolve during training and how action correction changes raw policy outputs into executable actions. To further examine the training behavior of learning-based allocation methods, Figure 3 compares PPO, PPO-Penalty, SAC, MAPPO, and the proposed CBL-CRL in terms of validation cost, CBL risk exposure, and constraint violation behavior. Unlike Table 3, which reports the final test-set performance under the normal scenario, this figure shows how different learning and constraint-handling strategies evolve during training. In Figure 3c, the violation rate denotes the fraction of demand response event periods in which an action violates at least one feasibility condition, including budget, price, capacity, or CBL risk constraints. For CBL-CRL, both the raw policy output before action correction and the corrected action after action correction are reported. Here, CBL-CRL raw denotes the direct output of the learned policy, while CBL-CRL corrected denotes the action actually dispatched after the feasibility correction module.

As shown in Figure 3a, all methods reduce validation cost as training proceeds, but their convergence behavior differs. PPO and PPO-Penalty start from similar validation costs because they share the same policy structure, while the penalty terms mainly affect the learning trajectory after policy updates. SAC and MAPPO converge to lower validation costs than PPO-based baselines, indicating the benefit of stronger continuous-control learning and multi-agent coordination. CBL-CRL starts with a relatively conservative validation cost due to action correction and CBL risk control, but it gradually catches up during training and stabilizes at the lowest validation cost. This result indicates that incorporating CBL credibility and feasibility correction does not prevent the policy from learning economically effective allocation decisions.

Figure 3b shows the evolution of CBL risk exposure. PPO, SAC, and MAPPO reduce CBL risk during training, but their final risk levels remain relatively high because they do not explicitly treat baseline credibility as a feasibility-related allocation factor. PPO-Penalty achieves lower CBL risk exposure than PPO by adding CBL-related penalty terms to the reward function, confirming that reward penalties can mitigate baseline-related allocation risk to some extent. However, its risk level remains higher than that of CBL-CRL. For CBL-CRL, the CBL risk exposure is evaluated on the corrected actions, since only corrected actions are dispatched. The lower and more stable risk trajectory suggests that using CBL credibility in both resource profiling and action correction is more effective than representing CBL risk only through soft reward penalties.

Figure 3c further distinguishes raw policy feasibility from post-correction feasibility. PPO, SAC, and MAPPO gradually reduce their violation rates as the learned policies become more stable, while PPO-Penalty further reduces violations by penalizing infeasible decisions in the reward function. Nevertheless, these baselines still retain nonzero violation rates after convergence. For CBL-CRL, the raw policy violation rate decreases during training as the policy learns to produce actions closer to the feasible region. This shows that the policy itself becomes more compatible with the operational constraints over time.

The corrected CBL-CRL action maintains a zero violation rate throughout training because the action correction module projects the raw policy output into the feasible set before dispatch. Therefore, the zero corrected-action violation rate should not be interpreted as evidence that the raw policy is always feasible. Instead, it shows that the implemented allocation satisfies the defined feasibility conditions after correction. The gap between the raw and corrected CBL-CRL curves also indicates that action correction is most important in the early training stage and becomes less frequently needed as the raw policy improves. Together, Table 3, and Figure 2 and Figure 3, provide complementary evidence: Table 3 summarizes overall performance, Figure 2 shows typical-day operation, and Figure 3 explains the learning and feasibility behavior behind the deployed results. Overall, Figure 3 shows that CBL-CRL combines learning-based improvement in raw decisions with explicit feasibility correction before implementation. The module-level contribution of action correction is further isolated in the ablation study.

### 4.3. Ablation Study

This subsection evaluates the contribution of the key components in the proposed CBL-CRL method under the normal scenario. Four variants are compared. CRL-noCBL retains the budget, price, and capacity constraints, but removes customer baseline credibility from user resource profiling and removes the CBL risk constraint from action correction. CBL-CRL w/o CBL-risk constraint keeps customer baseline credibility in user profiling, but removes only the CBL risk constraint from action correction. CBL-CRL w/o correction keeps the same profiling and policy structure as CBL-CRL, but removes the action correction module and retains only basic numerical clipping and allocation normalization. CBL-CRL is the complete proposed method.

Here, Vio denotes the fraction of demand response event periods in which the candidate action violates at least one feasibility condition, including budget, price, capacity, or CBL risk constraints. For CBL-CRL, the candidate action is the corrected action after action correction. In addition to the raw normalized total cost $C^{raw}$, the deployment-aware cost $C^{dep}$ is reported to evaluate the cost after infeasible candidate actions are handled by a conservative fallback.

As shown in Table 4, removing customer baseline credibility leads to the highest CBL risk exposure. Compared with the complete CBL-CRL method, CRL-noCBL increases $E_{CBL}$ from 0.087±0.009 to 0.161±0.014 and increases $E_{track}$ from 0.108±0.010 to 0.132±0.012. Its raw cost also increases from 0.897±0.006 to 0.910±0.008, and the deployment-aware cost further rises to 0.919±0.009. These results indicate that excluding baseline credibility weakens both response allocation quality and settlement-related reliability. Although CRL-noCBL still retains the basic operational constraints, it cannot distinguish whether the response assigned to a user group can be credibly measured. As a result, the learned policy may still allocate response tasks to groups with attractive capacity or cost but higher baseline-related uncertainty.

The comparison between CRL-noCBL and CBL-CRL w/o CBL-risk constraint shows that customer baseline credibility is useful even when it is only introduced into user profiling. CBL-CRL w/o CBL-risk constraint reduces $E_{CBL}$ from 0.161±0.014 to 0.119±0.012, reduces $E_{track}$ from 0.132±0.012 to 0.126±0.011, and slightly lowers the raw cost from 0.910±0.008 to 0.906±0.007. This means that resource profiling with CBL credibility can guide the agent toward user groups with more reliable response measurement. However, this variant still has a higher $E_{CBL}$, a higher Vio, and a higher $C^{dep}$ than the complete method. This suggests that using CBL credibility only as a profiling feature is helpful but incomplete; to control baseline-related allocation risk more effectively, CBL credibility should also enter the feasibility correction stage.

The effect of action correction is reflected by the comparison between CBL-CRL w/o correction and the complete CBL-CRL method. Without action correction, this variant achieves a relatively low raw cost of 0.902±0.007 and a tracking error of 0.116±0.013. These values suggest that feature-level CBL information can still guide the policy toward useful allocation decisions. However, its violation rate increases to 0.184±0.018, and its deployment-aware cost rises to 0.925±0.010. This indicates that the uncorrected policy can obtain seemingly competitive raw performance by selecting candidate actions that are not always executable. Therefore, the raw cost of this variant should not be interpreted alone; after infeasible actions are handled under deployment-aware evaluation, the practical value of action correction becomes clearer.

The complete CBL-CRL method achieves the best deployable performance across the evaluated metrics. Compared with CBL-CRL w/o CBL-risk constraint, it further reduces $C^{raw}$ from 0.906±0.007 to 0.897±0.006, $E_{track}$ from 0.126±0.011 to 0.108±0.010, and $E_{CBL}$ from 0.119±0.012 to 0.087±0.009. Compared with CBL-CRL w/o correction, it avoids the increase from raw cost to deployment-aware cost and removes violations in candidate actions. These results indicate that customer baseline credibility improves resource evaluation, while action correction converts the learned allocation into an executable decision under budget, price, capacity, and settlement-related constraints.

### 4.4. CBL Risk Constraint, Credibility Parameter, and Grouping Sensitivity

The CBL risk threshold $\epsilon_{\mathrm{CBL}}$ controls the maximum allowable baseline-related allocation risk in the action correction module. A smaller threshold makes the corrected allocation more conservative by limiting the response share assigned to user groups with lower baseline credibility. A larger threshold relaxes this restriction and provides a wider feasible allocation space. Therefore, $\epsilon_{\mathrm{CBL}}$ directly affects the trade-off between operational flexibility and settlement-related risk. The default value used in the main comparison is $\epsilon_{\mathrm{CBL}}=0.10$.

**Figure 4:**
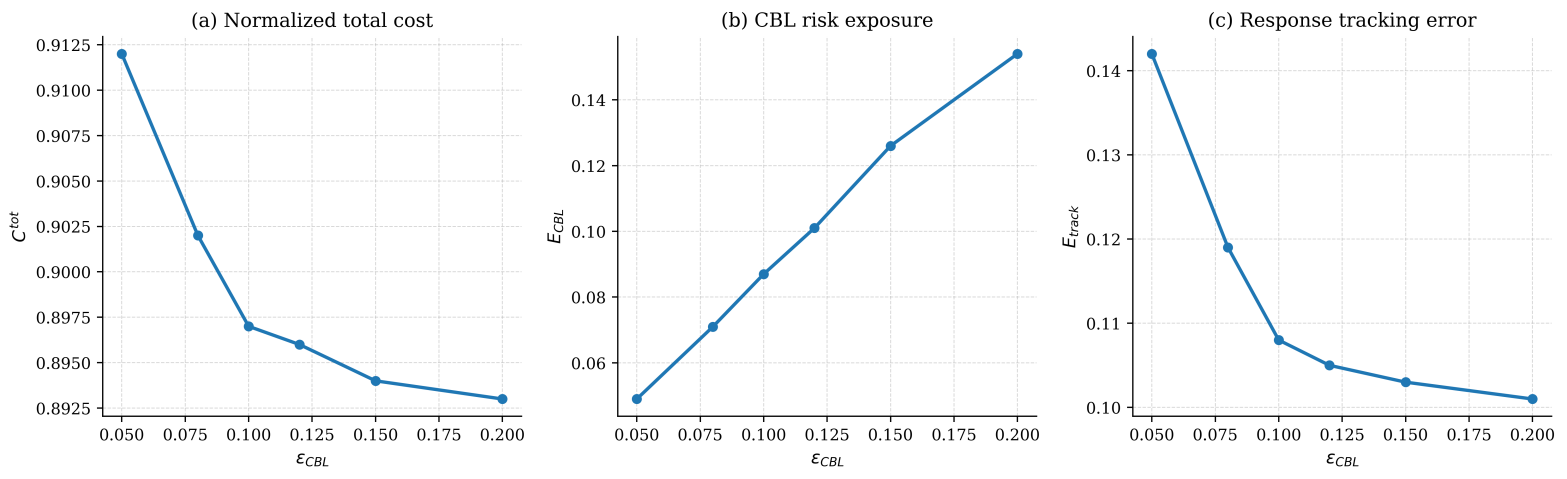
Sensitivity analysis of the CBL risk threshold. (**a**) Normalized total cost. (**b**) CBL risk exposure. (**c**) Response tracking error.

As shown in Figure 4a, the normalized total cost decreases as $\epsilon_{\mathrm{CBL}}$ increases. When $\epsilon_{\mathrm{CBL}}=0.05$, the CBL risk constraint is strict, and the policy relies more on high-credibility resources. This reduces the available response flexibility and leads to a higher total cost. When the threshold is relaxed from 0.05 to 0.10, the total cost decreases noticeably. Further increasing $\epsilon_{\mathrm{CBL}}$ from 0.10 to 0.20 only brings a marginal additional cost reduction, indicating that the economic benefit of relaxing the CBL risk constraint gradually diminishes.

Figure 4b shows the opposite trend for CBL risk exposure. As $\epsilon_{\mathrm{CBL}}$ increases, $E_{\mathrm{CBL}}$ rises from 0.049 to 0.154. This is expected because a looser threshold allows the corrected action to assign more response tasks to user groups with lower baseline credibility. Thus, although a larger $\epsilon_{\mathrm{CBL}}$ can slightly improve operating cost, it also increases baseline-related settlement risk.

Figure 4c shows that the response tracking error decreases as the CBL risk threshold becomes less restrictive. A strict threshold limits the feasible allocation space and may prevent the policy from assigning enough response tasks to meet the target response. After $\epsilon_{\mathrm{CBL}}$ reaches around 0.10–0.12, the tracking error decreases only slowly, while CBL risk exposure continues to increase. This indicates that most of the response tracking improvement has already been achieved in the moderate-threshold range.

Overall, the threshold sensitivity analysis reveals a clear trade-off between operational performance and baseline-related settlement risk. A very small $\epsilon_{\mathrm{CBL}}$ leads to conservative allocation and higher cost, whereas a very large threshold increases the probability of assigning response tasks to users with less credible baselines. In the tested case, $\epsilon_{\mathrm{CBL}}=0.10$–0.12 provides a practical trade-off range. Values around 0.10 offer stronger CBL risk control, while values around 0.12 slightly improve cost and tracking performance at the expense of higher baseline-related risk.

In addition to the CBL risk threshold, we further examine the sensitivity coefficient η in the CBL credibility mapping. Different from $\epsilon_{\mathrm{CBL}}$, which limits the maximum allowable allocation risk during action correction, η affects the user-level credibility scores before resource profiling and grouping. To verify its influence, CBL-CRL is tested under five η settings: $0.50\eta_{0}$, $0.75\eta_{0}$, $1.00\eta_{0}$, $1.25\eta_{0}$, and $1.50\eta_{0}$. Here, $\eta_{0}$ denotes the default value used in the main comparison. For each η setting, CBL credibility scores are recalculated, user groups are reconstructed, and the CBL-CRL policy is retrained and evaluated under the same user pool, event-triggering rule, budget level, $\epsilon_{\mathrm{CBL}}$, training protocol, and random seeds.

**Figure 5:**
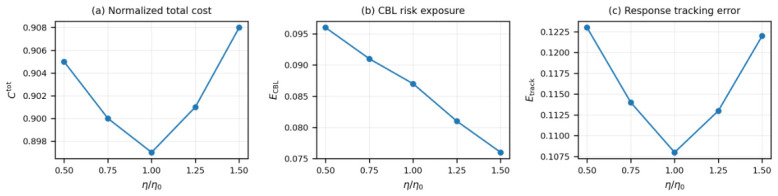
Sensitivity analysis of the CBL credibility sensitivity coefficient η. (**a**) Normalized total cost. (**b**) CBL risk exposure. (**c**) Response tracking error.

As shown in Figure 5, the η setting affects the trade-off among operating cost, response tracking, and CBL risk exposure. When $\eta =0.50\eta_{0}$, the credibility mapping is relatively relaxed. This setting enlarges the usable response resource set, but it also assigns more response responsibility to users with moderate baseline errors, leading to higher CBL risk exposure and response tracking error. Increasing η from $0.50\eta_{0}$ to $1.00\eta_{0}$ reduces the normalized total cost from 0.905 to 0.897, reduces CBL risk exposure from 0.096 to 0.087, and reduces response tracking error from 0.123 to 0.108.

When η continues to increase beyond the default value, CBL risk exposure keeps decreasing because baseline errors are penalized more strongly. Specifically, $E_{\mathrm{CBL}}$ decreases from 0.087 at $1.00\eta_{0}$ to 0.081 at $1.25\eta_{0}$ and 0.076 at $1.50\eta_{0}$. However, the stronger penalty also makes the allocation more conservative. As a result, the normalized total cost increases from 0.897 to 0.901 and 0.908, while the response tracking error increases from 0.108 to 0.113 and 0.122. These results indicate that an overly large η can reduce settlement-related risk, but may also restrict the available allocation space and weaken operational performance.

Therefore, η and $\epsilon_{\mathrm{CBL}}$ play complementary roles in the proposed framework. The sensitivity coefficient η shapes the credibility evaluation before resource grouping, while $\epsilon_{\mathrm{CBL}}$ limits the group-level allocation risk before dispatch. In the tested case, the default setting $1.00\eta_{0}$ provides the best balance among normalized total cost, response tracking error, and CBL risk exposure. This result supports the use of η as a practical risk-preference parameter.

To examine whether the proposed method depends on the default three-group structure, we further test CBL-CRL with different numbers of user groups. The tested settings include K=2, K=3, K=4, and K=5. For each setting, users are sorted by the resource quality score and divided into *K* groups using percentile-based thresholds. The policy action dimension becomes 2K, because each group requires one incentive multiplier and one response allocation ratio. All other settings, including the user pool, event-triggering rule, budget level, training length, and random seeds, are kept unchanged.

As shown in Table 5, the number of user groups controls the trade-off between allocation granularity and decision complexity. When K=2, the action dimension is low, but users with different reliability, response cost, and CBL credibility are merged into coarser resource classes. This leads to higher normalized total cost, response tracking error, and CBL risk exposure.

Increasing the group number from K=2 to K=3 improves all three performance metrics, reducing $C^{\mathrm{tot}}$ from 0.906 to 0.897, $E_{\mathrm{track}}$ from 0.124 to 0.108, and $E_{\mathrm{CBL}}$ from 0.112 to 0.087. Further increasing *K* to four and five slightly reduces $E_{\mathrm{CBL}}$ because user groups become more fine-grained, but the normalized total cost and response tracking error increase. The online decision time also increases from 0.33 ms at K=3 to 0.38 ms and 0.44 ms at K=4 and K=5, respectively.

These results indicate that the proposed method does not rely on an arbitrary grouping structure. The default setting K=3 provides the best balance in the tested case because it captures priority, regular, and conservative response resources while keeping the action dimension compact. Therefore, the three-group setting is used as the default implementation in the main experiments.

### 4.5. Robustness Under Different Operating Scenarios

**Figure 6:**
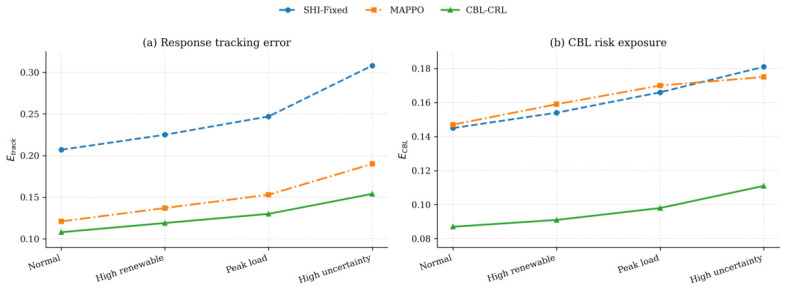
Robustness comparison under different operating scenarios. Panel (**a**) shows the response tracking error, and panel (**b**) shows the CBL risk exposure for SHI-Fixed, MAPPO, and CBL-CRL across four representative scenarios.

To evaluate the robustness of the proposed method under different operating conditions, this subsection compares SHI-Fixed, MAPPO, and CBL-CRL across four representative scenarios: normal, high renewable, peak load, and high uncertainty. SHI-Fixed is selected as the representative rule-based baseline, while MAPPO is selected as the strongest learning-based baseline according to the overall comparison in Section 4.1. Figure 6 reports the response tracking error and CBL risk exposure under these scenarios.

As shown in Figure 6a, all methods show higher response tracking error under stressed operating conditions than under the normal scenario. The high-uncertainty scenario leads to the largest degradation, indicating that user-side response uncertainty is the most challenging factor for accurate response tracking in this case study. SHI-Fixed has the highest tracking error in all scenarios because its fixed group-level incentives cannot adapt to time-varying system stress and user-side uncertainty. MAPPO substantially improves tracking performance over SHI-Fixed, confirming the value of adaptive learning-based allocation. CBL-CRL further reduces the tracking error to 0.108, 0.119, 0.130, and 0.154 under the four scenarios, respectively. Compared with MAPPO, the corresponding reductions are 10.7%, 13.1%, 15.0%, and 18.9%. These results indicate that CBL-CRL maintains more stable response tracking across the tested operating scenarios.

Figure 6b shows the corresponding CBL risk exposure. CBL-CRL maintains the lowest CBL risk exposure across all scenarios. In the normal scenario, its CBL risk exposure is 0.087, lower than 0.145 for SHI-Fixed and 0.147 for MAPPO. Under high-renewable, peak-load, and high-uncertainty scenarios, the CBL risk exposure of CBL-CRL increases moderately to 0.091, 0.098, and 0.111, respectively. Compared with MAPPO, the reductions in CBL risk exposure are 40.8%, 42.8%, 42.4%, and 36.6%. This indicates that the proposed method can preserve baseline-related settlement reliability even when renewable variability, load stress, or user response uncertainty increases.

The comparison between SHI-Fixed and MAPPO provides an additional observation. MAPPO consistently improves response tracking over SHI-Fixed, but it does not consistently reduce CBL risk exposure. In the normal, high-renewable, and peak-load scenarios, MAPPO has slightly higher CBL risk exposure than SHI-Fixed, while its CBL risk becomes lower than SHI-Fixed only under the high-uncertainty scenario. This suggests that a learning-based policy focused mainly on operational performance may allocate more response tasks to user groups with weaker baseline credibility. Therefore, adaptive learning improves dispatch performance, but it does not automatically improve settlement-related reliability.

Overall, the robustness results show that CBL-CRL maintains a better balance between response tracking and CBL risk control across the tested scenarios. The method still degrades under high uncertainty, which indicates that user-side uncertainty remains a challenging factor. However, its degradation is smaller than that of SHI-Fixed and MAPPO in both tracking error and baseline-related risk. This suggests that combining CBL credibility in resource profiling with action correction improves the robustness of incentive allocation under different operating conditions.

### 4.6. Discussion

The results indicate that the advantage of CBL-CRL comes from the joint use of dynamic group-level allocation, customer baseline credibility, and action correction. Rule-based strategies can activate part of the available flexibility, but their fixed incentive multipliers cannot adapt to event severity, remaining budget, or time-varying user-group conditions. Standard learning-based methods improve dynamic allocation, but they do not explicitly distinguish whether the delivered response can be credibly measured and settled. PPO-Penalty partly addresses this issue by adding constraint-related penalties to the reward function, but the results show that soft penalties still leave nonzero executed-action violations and higher CBL risk than CBL-CRL. Therefore, the proposed method improves allocation quality not by simply increasing response intensity, but by coordinating operating value, response measurability, and feasibility before execution.

A key implication is that demand response resources should not be ranked only by nominal flexible capacity. In practical incentive-based demand response programs, a user with large adjustable load may still be a low-quality resource if its response is unstable or its baseline is difficult to verify. By incorporating customer baseline credibility into resource profiling, the aggregator can distinguish between measurable flexibility and flexibility that may introduce settlement uncertainty. This design is particularly important for market-oriented demand response, where payment credibility and response verification affect long-term user participation. The results suggest that customer baseline credibility can serve as a pre-event allocation factor.

The action correction module further improves the feasibility of learned decisions. A reinforcement learning policy may generate actions that are attractive in terms of reward but infeasible under budget, capacity, price, or CBL risk constraints. In CBL-CRL, the raw policy output is corrected before dispatch, which explains the zero executed-action violation rate observed after action correction. This does not imply that the raw policy is always feasible; rather, it means that the implemented allocation is projected into the feasible set before execution. The CBL risk threshold sensitivity analysis further shows that the threshold should be treated as an operational design parameter. A stricter threshold provides stronger settlement-risk control but may increase cost and tracking error, while a looser threshold improves short-term flexibility at the expense of higher baseline-related risk.

The computational-time results further support online deployment. The training stage is performed offline, while real-time operation only requires state construction, policy inference, and action correction. Since the online decision time of CBL-CRL is 0.33 ms per decision period in the tested setting, the computation can be completed well within an hourly demand response scheduling interval. Therefore, the proposed framework can be updated offline using accumulated records and then executed online with low decision latency.

Several aspects should be considered for practical deployment. First, the case study uses a public industrial demand response dataset with a limited number of real factories, and the expanded user pool is generated from empirical characteristics of these factories. Larger demand response datasets from more user types would help further validate scalability. Second, the user-side and system-side data come from separate public sources. For deployment in a specific region, the framework should be recalibrated using local user load records, system load, renewable generation, market prices, baseline rules, and contract or bidding information. Third, the response cost attribute is generated in normalized form because monetary response cost is not directly reported in the dataset. Future work should incorporate contract data or bidding records when available. Fourth, this paper treats the baseline estimator as an interchangeable component and focuses on the allocation value of baseline credibility. More advanced baseline estimation models can be integrated in future studies.

Future work can extend the proposed framework in three directions. First, CBL-CRL should be tested with larger and more diverse demand response datasets, including commercial buildings, residential aggregations, and virtual power plant portfolios. Second, the current allocation framework can be combined with online baseline estimation and anomaly detection, so that baseline credibility can be updated more frequently during repeated events. Asynchronous and privacy-preserving federated learning can also be explored for updating user profiles and CBL credibility when user data cannot be centrally shared [58,59]. Third, the proposed mechanism can be embedded into market-oriented settlement models, where incentive prices, user bids, response verification, and aggregator revenue are jointly considered. These extensions would further improve the practical applicability of CBL-CRL in real demand response programs.

## 5. Conclusions

This paper proposed a constrained reinforcement learning method with customer baseline credibility for dynamic resource allocation in incentive-based demand response. The main motivation was to move customer baseline load from a post-event settlement reference to a pre-event allocation factor. In the proposed framework, users are evaluated by flexible capacity, response reliability, response cost, and customer baseline credibility. The resulting user profiles are used to form resource groups, and the reinforcement learning agent determines group-level incentive multipliers and response task allocation ratios. An action correction module is further introduced to convert raw policy outputs into feasible allocation decisions under budget, price, capacity, and CBL risk constraints.

The case study results show that the proposed method improves both operational performance and settlement-related reliability. Under the normal scenario, CBL-CRL reduces the normalized total cost to 0.897±0.006, achieves the lowest response tracking error of 0.108±0.010, and reduces CBL risk exposure to 0.087±0.009. Compared with MAPPO, CBL-CRL further reduces CBL risk exposure from 0.147±0.019 to 0.087±0.009, while maintaining the same renewable accommodation rate of 0.970. Since the demand response task is triggered by high net-load stress, this renewable accommodation result should be interpreted as maintaining comparable curtailment-related system performance. The main advantage of CBL-CRL is its balanced improvement in cost, response tracking, feasibility, and baseline-related settlement reliability.

The learning and ablation results further clarify the role of the proposed components. PPO-Penalty shows that reward penalties can reduce CBL risk and feasibility violations to some extent, but they do not fully replace explicit action correction. The complete CBL-CRL method maintains a zero executed-action violation rate after action correction under the defined constraints, while still achieving lower validation cost and lower CBL risk exposure than the learning-based baselines. The ablation study also shows that customer baseline credibility is useful both in resource profiling and in action correction. Using baseline credibility only as a profiling feature is beneficial but insufficient for controlling baseline-related allocation risk.

The sensitivity and robustness analyses indicate that CBL-CRL remains effective under different operating conditions, although user-side uncertainty still increases both tracking error and CBL risk exposure. The CBL risk threshold is an important operational parameter. A very strict threshold may make the allocation overly conservative and increase cost, while a loose threshold may increase baseline-related settlement risk. In the tested case, the range of $\epsilon_{\mathrm{CBL}}=0.10$–0.12 provides a practical trade-off, where values around 0.10 offer stronger risk control and values around 0.12 provide slightly better cost and tracking performance at the expense of higher CBL risk. The added sensitivity analysis of η further shows that η controls the strictness of user-level CBL credibility evaluation.

Several directions remain for future work. First, larger and more diverse real-world demand response datasets should be used to further validate the scalability of the proposed method. Future studies can also use regionally matched user-side and system-side records to evaluate the framework under local operating conditions. Second, monetary response cost and user bidding information should be incorporated when such data are available, so that incentive allocation can be linked more directly to market behavior. Third, more advanced baseline estimation and anomaly detection models can be integrated into the current framework to improve the accuracy and robustness of CBL credibility evaluation. Finally, the proposed method can be extended to virtual power plants, multi-energy systems, and market-oriented settlement mechanisms, where flexible resources, response verification, and incentive payments need to be coordinated in a unified decision-making framework.

## Figures and Tables

**Table 1:** Implementation settings of user resource profiling and grouping.

Item	Setting	Allocation Meaning
Profiling attributes	$Cap_{i}$, $Rel_{i}$, $Cost_{i}$, and $q_{i}^{\mathrm{CBL}}$ are used after normalization.	Describe user flexibility, execution reliability, response cost, and baseline credibility.
Resource score	$S_{i}=w_{\mathrm{cap}}Cap_{i}+w_{\mathrm{rel}}Rel_{i}+w_{\mathrm{cbl}}q_{i}^{\mathrm{CBL}}-w_{\mathrm{cost}}Cost_{i}$.	Ranks users by dispatch value and settlement credibility.
Grouping rule	Users are sorted by $S_{i}$ and divided into A/B/C groups using percentile-based thresholds.	Converts heterogeneous users into allocation-oriented resource classes.
Group A	High-score users.	Priority response group.
Group B	Medium-score users.	Regular response group.
Group C	Low-score or risk-limited users.	Conservative response group under stricter allocation control.
Update rule	Profiles and groups are updated when new response records or baseline validation samples are available.	Supports dynamic resource management across repeated events.

**Table 2:** Key hyperparameters and experimental settings.

Category	Parameter	Setting
Data split	Train/validation/test	70%/15%/15%
Temporal setting	Decision period	1 h
Temporal setting	Episode length	24 periods
User pool	Number of users	50
Budget	Daily incentive budget	80% full-target payment
User grouping	Number of groups	K=3
Action space	Incentive multiplier	[0.6,1.5]
Action space	Allocation ratio	Softmax + correction
CBL risk control	Risk threshold	$\epsilon_{\mathrm{CBL}}$ = 0.10
Network	Actor / critic	2 layers, 128 neurons
Activation	Hidden activation	ReLU
Training	Learning rate	$3\times 10^{-4}$
Training	Discount factor	γ=0.99
PPO update	Clip parameter	0.2
Training	Mini-batch size	256
Training	Training length	300,000 steps
Robustness	Random seeds	5

**Table 3:** Overall performance and computational-time comparison under the normal scenario. The results are averaged over five random seeds. Standard deviations are reported for $C^{\mathrm{tot}}$, $E_{\mathrm{track}}$, $E_{\mathrm{CBL}}$, $T_{\mathrm{train}}$, and $T_{\mathrm{dec}}$, while the other metrics are reported as mean values. Arrows indicate whether a higher or lower value is preferred.

Method	$C^{\mathrm{tot}}↓$	$C_{\mathrm{inc}}↓$	$\eta_{\mathrm{RE}}↑$	$E_{\mathrm{track}}↓$	$\eta_{\mathrm{inc}}↑$	$E_{\mathrm{CBL}}↓$	$T_{\mathrm{train}}$ (min) ↓	$T_{\mathrm{dec}}$ (ms) ↓
*Reference*								
No-DR	1.000	0.000	0.941	1.000	N/A	N/A	–	–
*Rule-based methods*								
UNI-Cap	0.946±0.007	0.060	0.954	0.279±0.018	0.90	0.188±0.013	–	0.04±0.01
SHI-Fixed	0.929±0.006	0.053	0.961	0.207±0.016	1.34	0.145±0.015	–	0.06±0.01
*Learning-based methods*								
PPO	0.918±0.012	0.050	0.965	0.157±0.012	1.64	0.166±0.021	8.7±0.5	0.23±0.02
PPO-Penalty	0.916±0.017	0.052	0.966	0.150±0.014	1.62	0.132±0.016	9.2±0.6	0.24±0.03
SAC	0.912±0.008	0.048	0.967	0.139±0.015	1.83	0.158±0.018	12.6±0.8	0.31±0.03
MAPPO	0.905±0.009	0.047	0.970	0.121±0.011	2.02	0.147±0.019	15.8±0.9	0.46±0.04
*Proposed method*								
CBL-CRL	0.897±0.006	0.049	0.970	0.108±0.010	2.10	0.087±0.009	10.4±0.7	0.33±0.03

**Table 4:** Ablation study results under the normal scenario. The results are averaged over five random seeds. $C^{raw}$ denotes the raw normalized total cost, while $C^{dep}$ denotes the deployment-aware cost after replacing infeasible candidate actions with a conservative fallback. Arrows indicate whether a lower value is preferred for each metric.

Variant	$C^{\mathrm{raw}}↓$	$C^{\mathrm{dep}}↓$	$E_{\mathrm{track}}↓$	$E_{\mathrm{CBL}}↓$	Vio↓
CRL-noCBL	0.910±0.008	0.919±0.009	0.132±0.012	0.161±0.014	0.083±0.011
CBL-CRL w/o					
CBL-risk constraint	0.906±0.007	0.913±0.008	0.126±0.011	0.119±0.012	0.062±0.010
CBL-CRL w/o					
correction	0.902±0.007	0.925±0.010	0.116±0.013	0.104±0.011	0.184±0.018
CBL-CRL	0.897±0.006	0.897±0.006	0.108±0.010	0.087±0.009	0.000±0.000

Bold values indicate the best performance among the compared variants.

**Table 5:** Sensitivity analysis with different numbers of user groups. Arrows indicate whether a lower value is preferred for each metric.

*K*	Action Dim.	$C^{\mathrm{tot}}↓$	$E_{\mathrm{track}}↓$	$E_{\mathrm{CBL}}↓$	$T_{\mathrm{dec}}\;\left(\mathrm{ms}\right)↓$
2	4	0.906±0.008	0.124±0.013	0.112±0.012	0.29±0.03
3	6	0.897±0.006	0.108±0.010	0.087±0.009	0.33±0.03
4	8	0.899±0.007	0.111±0.011	0.083±0.009	0.38±0.03
5	10	0.904±0.009	0.118±0.012	0.080±0.010	0.44±0.04

## Data Availability

The data analyzed in this study are available from the publicly available South Korean manufacturing factories’ electricity consumption and demand response dataset and the Open Power System Data time-series dataset cited in the article.
